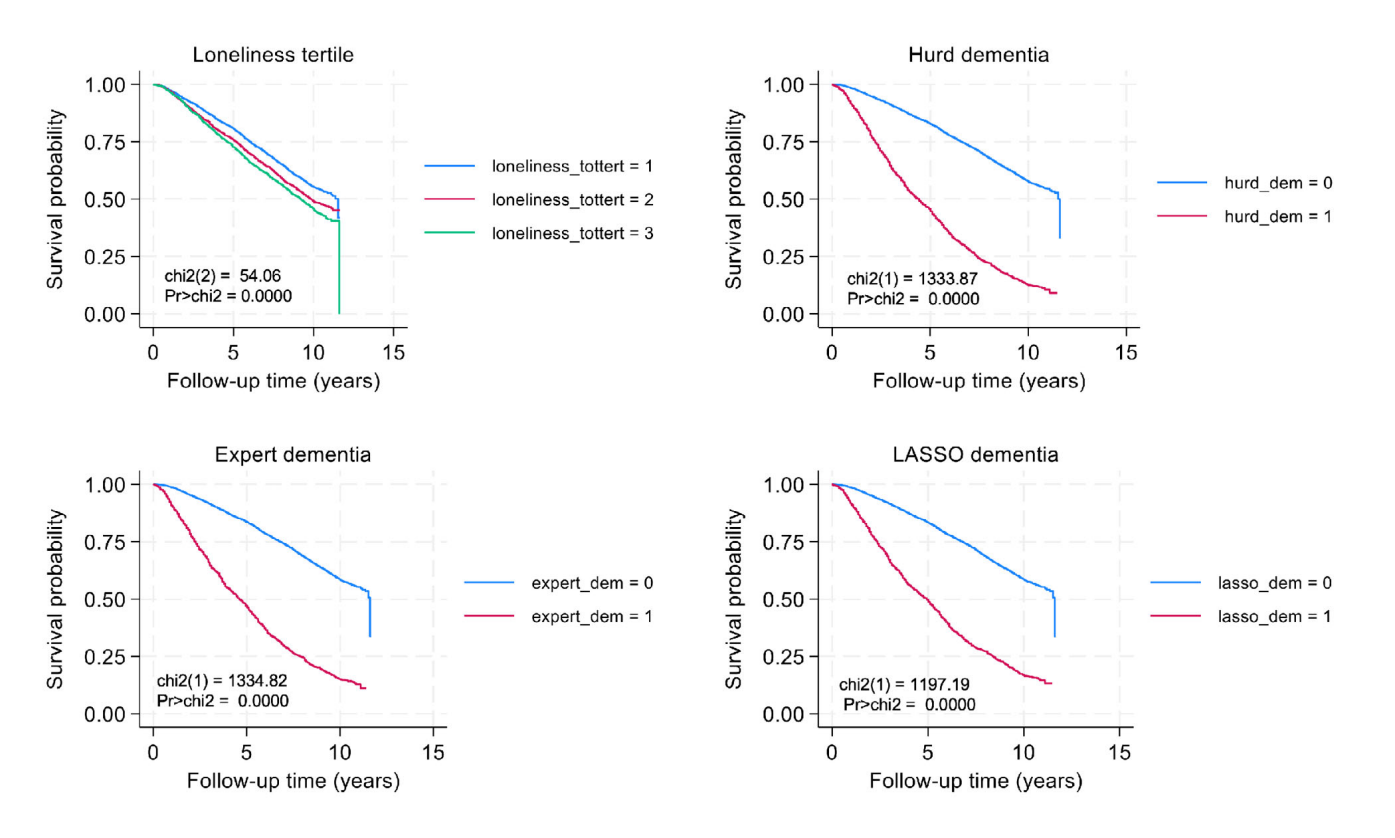# Loneliness, dementia status and their association with all‐cause mortality among older US adults

**DOI:** 10.1002/alz.089234

**Published:** 2025-01-09

**Authors:** Michael F Georgescu, May A Beydoun, Jason Ashe, Christian A Maino Vieytes, Hind A Beydoun, Michele K Evans, Alan B Zonderman

**Affiliations:** ^1^ NIA/NIH/IRP, Baltimore, MD USA; ^2^ National Institute on Aging, NIA/NIH/IRP, Baltimore, MD USA; ^3^ Laboratory of Epidemiology & Population Sciences, Baltimore, MD USA; ^4^ VA National Center on Homelessness Among Veterans, U.S. Department of Veterans Affairs, Washington, DC, DC USA

## Abstract

**Background:**

Loneliness, dementia, and mortality are interconnected. Thus, understanding the pathways, along with their potential interaction provides a relevant area of research to be developed.

**Method:**

The study tested bi‐directional relationships between dementia, loneliness and mortality, while examining both interactions and mediating effects in a large sample of older US adults participating in the nationally representative Health and Retirement Study. Out of ≤6,468 older participants selected in 2010, with mean baseline age of 78.3 y and a follow‐up time up to end of 2020, 3,298 died with a rate of 64 per 1,000 P‐Y. Cox proportional hazards models were used, stratifying by sex, race and interaction between loneliness scores (continuous or tertiles) and dementia status or Loge(odds) were examined. Four‐way decomposition models were also carried out to test bi‐directional mediation and interaction between dementia odds and loneliness in relation to mortality risk.

**Result:**

Probable dementia was consistently linked to over two‐folds increased mortality risk. The presence of dementia and Loge(odds) were equally strongly related with mortality risk across tertiles of loneliness score. The study also found that the loneliness z‐score was linked to an elevated risk of mortality regardless of age, sex, or race or ethnicity, and that its total effect (TE) on mortality was partially mediated by Loge odds of dementia (z‐scored), with up to 40% of TE being a pure indirect effect. In contrast, a very small proportion (<5%) of the TE of dementia odds (Loge transformed, z‐scored) on mortality risk was explained by the loneliness z‐score.

**Conclusion:**

transformed, z‐scored) on mortality risk was explained by the loneliness z‐score. Conclusions. In sum, dementia was positively associated with mortality risk, in similar fashion across loneliness score tertiles, while loneliness was associated with mortality risk in the overall sample. The total effect of loneliness on mortality risk was partially mediated by dementia probability, particularly when models were only adjusted for age, sex, and race/ethnicity.